# Cyclopropenium Nanoparticles and Gene Transfection in Cells

**DOI:** 10.3390/pharmaceutics12080768

**Published:** 2020-08-13

**Authors:** Noam Y. Steinman, Luis M. Campos, Yakai Feng, Abraham J. Domb, Hossein Hosseinkhani

**Affiliations:** 1 Institute of Drug Research, School of Pharmacy-Faculty of Medicine, The Hebrew University of Jerusalem, Jerusalem 91120, Israel; Noam.Steinman@mail.huji.ac.il; 2 Department of Chemistry, Columbia University, New York, NY 10027, USA; lc2730@columbia.edu; 3 School of Chemical Engineering and Technology, Tianjin University, Yaguan Road 135, Tianjin 300350, China; yakaifeng@tju.edu.cn; 4 The Alex Grass Center for Drug Design and Synthesis and Center for Cannabis Research and the Institute of Drug Research, School of Pharmacy-Faculty of Medicine, The Hebrew University of Jerusalem, Jerusalem 91120, Israel; 5 Innovation Center for Advanced Technology, Matrix, Inc., New York, NY 10029, USA; hh@matrix-inc.com

**Keywords:** cyclopropenium, cationic nanoparticles, gene transfection, poly(ethylene imine)

## Abstract

Non-viral vectors for the transfection of genetic material are at the frontier of medical science. In this article, we introduce for the first time, cyclopropenium-containing nanoparticles as a cationic carrier for gene transfection, as an alternative to the common quaternary ammonium transfection agents. Cyclopropenium-based cationic nanoparticles were prepared by crosslinking poly(ethylene imine) (PEI) with tetrachlorocyclopropene. These nanoparticles were electrostatically complexed with plasmid DNA into nanoparticles (~50 nm). Their cellular uptake into F929 mouse fibroblast cells, and their eventual expression in vitro have been described. Transfection is enhanced relative to PEI with minimal toxicity. These cyclopropenium nanoparticles possess efficient gene transfection capabilities with minimal cytotoxicity, which makes them novel and promising candidates for gene therapy.

## 1. Introduction

The introduction of nucleotide-based agents into cells for both therapeutic and experimental applications is an expanding field of research, particularly for the treatment of genetic diseases [1]. Many vectors, both viral and non-viral, have been developed as agents to transfect genes and express them in cells [2,3]. Poly(ethylene imine) (PEI) is a readily available polymer bearing primary, secondary and tertiary amine groups which may be protonated at low pH, forming a polycation [4]. This polycation has been used to condense plasmid DNA into stable polyplexes, enabling transfection via endocytosis [5]. The formation of nano-size polyplexes is crucial for transfection efficiency [6].

Successful transfection mediated by non-viral polymer vectors is a promising strategy as they can effectively condense DNA into small particles, protect DNA from enzymatic degradation, enter the cell by endocytosis, and dispose the payload into the nucleus [7,8,9]. Several chemical characteristics of PEI have been reported to contribute to its consideration as one of the most widely-used non-viral transfection vectors [10]. Its cationic charge allows polyplex formation with polyanionic exogenous DNA, thereby forming condensed particles which mask the phosphodiester bond from cleavage by DNase [11,12,13,14]. Endosomal release is facilitated by a proton sponge effect, whereby amines along the PEI chain protonate in the endosome amid increased endosomal pH [15], causing an influx of counter ions that increase osmotic pressure, disrupting the endosomal membrane [16,17]. The PEI/DNA polyplex is then able to escape the damaged endosome, leading to PEI/DNA decomplexation and expression of the DNA within the cell.

Although commonly used, PEI suffers from a change in efficiency related to changes in protonation of the amines along the polymer chain at varying pH [18,19,20]. To overcome this, quaternary ammonium compounds with pH-insensitive positive charges have been developed as transfection agents [21]. Indeed, even PEI has been outfitted with quaternary ammonium, endowing it with pH-independent positive charge [22]. While these materials increase transfection efficiency, quaternary ammonium compounds suffer from reduced chemical stability due to undesirable chemical reactions or thermal degradation [23,24]. In this study, a polymer-containing cyclopropenium, a positively charged aromatic moiety, was introduced as a potential gene transfection agent.

Previous studies with permanently charged polycationic systems based on tris-aminocyclopropenium (TAC) cations have shown remarkable transfection efficiencies from linear polymers, micelles, and nanoparticles [25,26,27]. The TAC cation remains positively charged over a wide range of pH values (~2 to ~13) [28,29]. Taking into consideration the advantages of using the TAC cation with the pH-responsive PEI system, we sought to develop PEI nanoparticles that are randomly crosslinked with TAC moieties throughout. These materials are expected to enhance the transfection capabilities of PEI, as the permanently cationic TAC may prevent premature dissociation of DNA, a limiting factor in polyplexes based on PEI alone [30]. Moreover, we posit that the increased cationic charge of the polyplex may enhance endosomal escape by increasing the disruption of the vesicular membrane. It is also worth noting that the synthesis of PEI/TAC-based nanoparticles is relatively straightforward, from commercially available substrates. The synthesis is typically carried out by the instant, high yield reaction between the TAC precursor, tetrachlorocyclopropene (TCC), and secondary amines [31]. In this report, we demonstrate the formation of cyclopropenium-containing nanoparticles that efficiently form polyplexes with plasmid DNA, exhibiting increased cellular uptake and transfection over quaternary ammonium-based transfection molecules.

## 2. Materials and Methods

### 2.1. General Crosslinking Procedure

Poly(ethylene imine) (PEI) 25 kDa linear, 5 kDa linear, and 1.3 kDa branched, and tetrachlorocyclopropene (TCC) were purchased from Sigma-Aldrich (Rehovot, Israel). Chloroform was purchased from Bio-Lab, Ltd. (Jerusalem, Israel). Crosslinking was performed as reported previously [32]. Briefly, TCC (10–60 mol%) was added dropwise to a 0.5 mM solution of PEI in chloroform at 0 °C. A white precipitate formed instantly, and the reaction was left to warm to room temperature and stirred overnight. The solid was then isolated via filtration and dried under vacuum to yield cyclopropenium-crosslinked PEI (PEI/TAC).

### 2.2. Complexation of Cyclopropenium Polymer Nanoparticles with DNA

All studies were performed on PEI/TAC with unmodified PEI as a reference. A plasmid DNA (12.5-kb DNA) containing a cytomegalovirus (CMV) promoter coding the firefly luciferase was used in this study. Complexation of the polymer with DNA was performed by mixing the two materials at weight ratios of 2:1, 5:1 and 10:1 (polymer/DNA) in phosphate-buffered saline (PBS) (2, 5 or 10 µg polymer and 1 µg DNA in 150 µL PBS). The solution was gently agitated at 37 °C for 30 min to form polymer/DNA polyplexes.

### 2.3. Cytotoxicity of Polyplexes

Cytotoxicity of polymer/DNA polyplexes was investigated in cultured F929 mouse fibroblast cells. The cells were plated in 96-well culture plates at a density of 10^3^ cells/well. F929 mouse fibroblast cells were cultured in RPMI-1640 supplemented with 10% (*v*/*v*) fetal bovine serum (FBS) and 1% (*v*/*v*) penicillin-streptomycin. Polymer/DNA polyplexes were then introduced to the cell culture medium at 37 °C. After 24 h, media were removed and 100 μL of fresh medium and 13 μL of MTT solution (5 μg/mL, diluted with RPMI 1640 without phenol red) were added. Incubation was allowed for another 4 h in the dark at 37 °C. Media were removed and 100 μL/well of 0.04 M HCl in isopropanol was added to dissolve the formazan crystals. The wells were read at 570 nm using an ELISA plate reader (BioTek Instruments, Inc., Winooski, VT 05404, USA), and cell viability (defined as 100% for MTT assay control) was calculated.

### 2.4. Cellular Uptake of Polyplexes into Cells

Plasmid DNA and rhodamine isothiocyanate (RITC) were mixed in 0.2 M sodium carbonate-buffered solution (pH 9.7) at 4 °C for 12 h at 1 mg/mL. The reaction mixture was applied to gel filtration with a PD 10 column to separate the RITC-labelled plasmid DNA from the uncoupled RITC reagent, followed by ethanol precipitation to obtain a RITC-labelled DNA. The RITC-labelled plasmid DNA was mixed with PEI/TAC nanoparticles at 2:1, 5:1 and 10:1 polymer/plasmid DNA weight ratios to prepare polyplexes that were added to each well in which L929 mouse fibroblast cells were grown at 70% confluence. The cells were incubated for 2 days, washed with PBS (3 × 1 mL) and lysed by 500 μL of a lysis buffer. The fluorescence intensity of cell lysates was measured by a fluorescent spectrophotometer (ex 570 nm/em 595 nm) and divided by that of RITC-labelled plasmid DNA initially added to obtain the percent internalized.

### 2.5. Transfection by Polymer/DNA Polyplexes

L929 mouse fibroblast cells were cultured in Dulbecco’s modified eagle’s medium supplemented with 10 wt% fetal calf serum, 0.12 wt% sodium bicarbonate, and 100 units/mL mixed penicillin-streptomycin solution. The cell suspension (10^5^ cells/2 mL) was plated into each well of a 6-well culture plate. The polyplex was added to each well once cell confluence reached 70%. After incubation for 4 h, the culture medium containing polyplexes was excluded and fresh medium (2 mL) was added. Cells were then incubated for an additional 48 h, washed 2× with 1 mL PBS, and lysed by 100 μL of lysis buffer. The cell lysate was centrifuged at 12,000 rpm for 5 s at 4 °C, and the supernatant was carefully collected and kept in ice. A supernatant sample (16 μL) was mixed with 80 μL of reconstituted luciferase assay solution, and the relative light unit (RLU) of the solution mixture was determined by a luminometer (Lumat Lb 9507, Berthold, Germany).

## 3. Results and Discussion

### 3.1. Preparation of Cyclopropenium-Crosslinked Poly(ethylene imine) (PEI)

**Figure 1:**
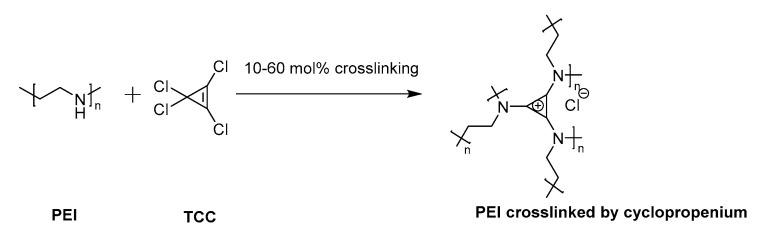
Cyclopropenium was generated as a chemical crosslinker of poly(ethylene imine) (PEI) by reaction with precursor tetrachlorocyclopropene (TCC).

The development of non-viral vectors for efficient gene transfection is at the frontier of therapy for genetic diseases [33]. The relatively newfound possibility of providing a cell with plasmid nucleic acids, without generating a significant immune response, has encouraged many to search for efficient and safe methods. PEI has emerged as an industry leader that is readily accessible, inexpensive, and an easy to handle polymer for the transfection of genetic material. In order to improve upon the transfection efficiency of PEI, a series of PEI nanoparticles was prepared by crosslinking with trisaminocyclopropenium (TAC). In a previous study [32], we showed that this crosslinking procedure provided cyclopropenium-crosslinked PEI nanoparticles upon dispersion into aqueous media (Figure 1). We confirmed the presence of the cyclopropenium ring in the precipitate with 14c NMR and FTIR spectroscopy. An additional signal in the 14c NMR spectra of crosslinked PEI compared to parent PEI was observed at a chemical shift of 170 ppm, indicating the presence of the C=C bonds of the aromatic ring. The formation of the cyclopropenium ring was also confirmed by the disappearance of N–H absorbances in the FTIR spectra at ~2900 cm^−1^. Thermal gravimetric analysis showed that crosslinked polymers displayed higher stability at temperatures below 300 °C. Representative data for these materials are available as Supplementary Materials (Figures S1 and S2).

**Figure 2:**
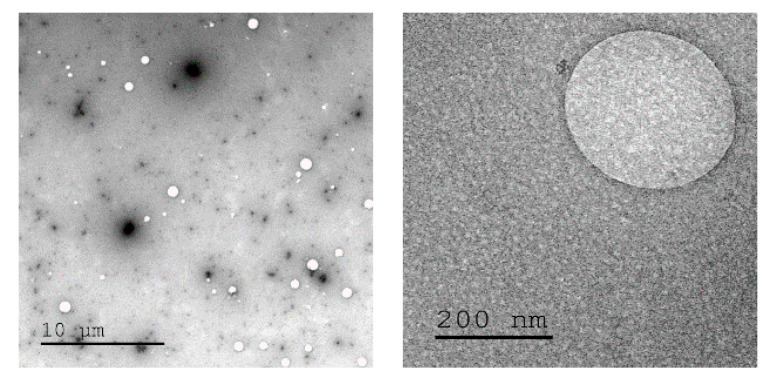
Transmission electron microscope images of PEI/trisaminocyclopropenium (TAC) nanoparticles in aqueous media.

The concept of crosslinking PEI with cyclopropenium was applied here as cationic non-viral transfection agents. Nanoparticles were prepared at various ratios between linear PEI of molecular weight (MW) 25 kDa (L-PEI 25k) and TAC (10, 30, or 60% amine conjugation with TAC, Figure 1). This particular PEI was selected due to its widespread use as a relatively efficient non-viral transfection agent [34]. Several degrees of crosslinking were prepared to study the effects of increased overall TAC content. Cyclopropenium was employed in order to improve upon the transfection efficiency of PEI by targeting both the internalization and endosomal escape steps of its mechanism of action. Furthermore, previous studies have shown that PEI nanoparticles may display enhanced transfection due to their condensed size [35]. Moreover, due to the pH-independent positive charge of TAC, the polyplex should form in high efficiency even under the relatively non-acidic conditions of the extracellular space [25]. This was seen as an especially critical advantage, since efficient transfection by PEI requires use of a large excess of polymeric material to form polyplexes with DNA; many of the amine groups along the polymer chain may not be protonated and, thereby, without charge. The cationic PEI/TAC nanoparticle, however, maintains its cationic nature at all relevant pH values [32], and so it is more readily available to bind the genetic material. Some amine groups along the polymer chain were left without crosslinking to maintain PEI’s ability to increase cationic charge only once in the endosome, which eventually leads to the rupture of the membrane and the release of the polyplex into the cell. (PEI/TAC) were isolated as insoluble white powders and were evaluated relative to commercial L-PEI 25k. PEI/TAC condensed into nanoparticles (~100 nm hydrodynamic diameter) with positive surface charge upon dispersion in aqueous media (Figure 2 and Table 1).

### 3.2. Polyplexes of PEI/TAC and Plasmid DNA

One of the motivations for employing polycations as non-viral gene transfection vectors is their ability to form a polyplex with polyanionic DNA [36]. Generally, transfection efficiency is significantly improved by the small particle size of the carrier:DNA complex (<200 nm), as smaller particles tend to enhance endocytosis [34]. When crosslinking PEI with TAC, the greater cationic charge of crosslinked polymers allowed for the collapse into nanoparticles (~100 nm) with dispersion into aqueous media, even before polyplex formation (Table 1).

Polyplexes were formed between PEI/TAC and a DNA construct (12.5-kb DNA) containing a cytomegalovirus (CMV) promoter inserted at the upstream region of sequence coding the firefly (*Photinus pyralis*) luciferase that had been isolated from bacteria. To do this, PEI/TAC and DNA were mixed into PBS solution to form polymer/DNA polyplexes.

**Figure 3:**
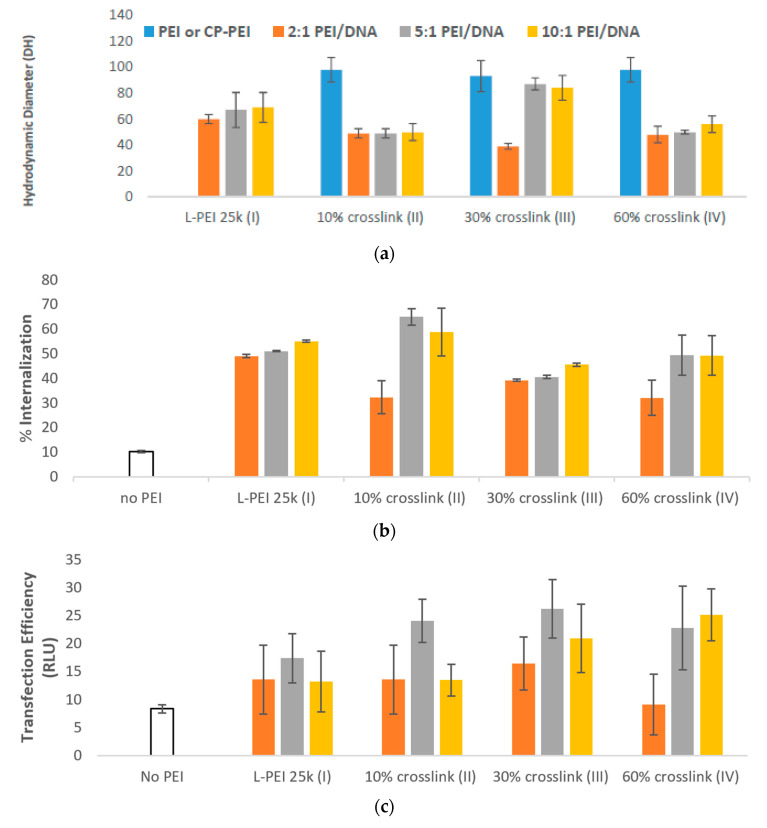

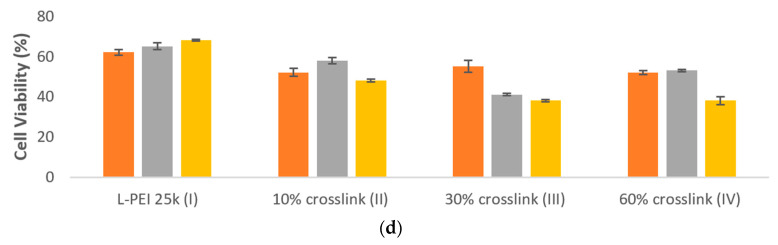
(**a**) Formation of PEI/TAC/DNA polyplexes was confirmed by the reduction in hydrodynamic diameter of particles. Both unmodified PEI and PEI/TAC formed polyplexes with DNA, allowing for cellular uptake by endocytosis; (**b**) Internalization of DNA was improved by PEI/TAC vectors. Crosslinking over 10% of the amine groups afforded no benefit compared to unmodified PEI; (**c**) Transfection efficiency was quantified by the relative light unit (RLU) of the transfected luciferase gene. PEI/TAC polymers displayed a marked enhancement of gene expression in vitro; (**d**) Cell viability of PEI/DNA polyplexes. MTT assay control was defined as 100%. PEI/TAC nanoparticles are not significantly more toxic than widely-used unmodified PEI.

The optimal mixing ratio of PEI/TAC and DNA was evaluated by mixing the DNA with PEI/TAC at various weight ratios (2:1, 5:1, 10:1 PEI/TAC:DNA). Particles formed with PEI/TAC were generally smaller than those prepared with unmodified PEI, indicating a more condensed particle (Figure 3a). Smaller polyplex particle sizes were expected to positively influence cellular uptake. Surprisingly, increased degrees of crosslinking did not drastically alter polyplex size. In most cases, the hydrodynamic diameter of the particle was reduced upon PEI/TAC/DNA complexation (~50 nm), indicating polyplex formation. L-PEI 25k condensed DNA to 60–70 nm (Figure 3a).

### 3.3. Internalization and Transfection

Cellular uptake of the PEI/TAC/DNA polyplexes was measured for polyplexes formed with DNA labelled by rhodamine isothiocyanate. Internalization of polyplexes was quantified as a percentage of labelled DNA to enter the cell by fluorescence of L929 mouse fibroblast cell lysates after two days of mixing with the polymer/DNA polyplex.

PEI/TAC afforded enhancement of DNA cellular uptake. When using unmodified PEI, a maximum of 55% of DNA was brought into the cell, requiring a 10:1 *w*/*w* mixing ratio of PEI/DNA (Figure 3b). At a 5:1 *w*/*w* mixing ratio (requiring half of the amount of polymer), polyplexes with PEI/TACs III and IV (30 and 60% crosslinking of L-PEI 25k, respectively) displayed only minor enhancements to internalization. PEI/TAC II, with only 10% of the amines crosslinked by TAC, however, succeeded in internalization of 65% of DNA at the 5:1 *w*/*w* mixing ratio.

Transfection of DNA was measured by a similar procedure. Cells were exposed to PEI/TAC/DNA polyplexes comprised of luciferase-coding DNA for 4 h to allow uptake, followed by 48 h incubation to allow for transfection. Cells were then lysed after washing twice with fresh PBS to remove unincorporated polyplexes, and mixed with a reconstituted luciferase assay solution. The relative light unit (RLU) of the solution mixture was determined by a luminometer to determine transfection efficiency.

Despite minor enhancements in internalization efficacy by PEI/TAC relative to unmodified PEI, crosslinking by TAC did enhance the overall transfection efficiency of the polymer. For unmodified PEI, the maximum transfection (by luciferase assay) was 17 RLU, achieved by a 5:1 *w*/*w* mixing ratio. Indeed, transfection efficiency was maximized at the 5:1 *w*/*w* mixing ratio for all polymers used. Maximum transfection was achieved by III (30% crosslinking) at this optimized mixing ratio with overall transfection of 26 RLU. Transfection by II (10% crosslinking) and IV (60% crosslinking) afforded similarly enhanced 24 and 23 RLU, respectively (Figure 3c). Considering the negligible effects on internalization, the increase in overall transfection efficiency when PEI/TAC was used may be attributed to enhanced endosomal escape.

These results support our hypothesis, as we suspected that the crosslinking of PEI with TAC would enhance transfection based on the reported mechanism of gene transfection by cationic polymers. Specifically, we expected that an increased cationic charge of the polymer would successfully bind more genetic material, as well as promote endosomal escape which occurs due to the proton sponge effect of cationic PEI. Indeed, the cationic strength afforded by crosslinking with TAC successfully enhanced the transfection capabilities of PEI, a result previously unattained by TAC-containing polymers [25].

### 3.4. Cytotoxicity

Cationic polymers are known to be cytotoxic, inducing apoptosis due to interactions with, and disruption of, negatively charged cell membranes [37,38,39]. The amount of polymer used in any biomedical application should, therefore, be minimized. Nevertheless, PEI (especially L-PEI 25k) is widely considered the gold standard of non-viral transfection agents, as its transfection efficacy may outweigh concerns about its toxicity [14,40].

PEI/TAC polyplexes were evaluated for cytotoxicity with F929 mouse fibroblast cells using MTT assay after 24 h of exposure. Whereas polyplexes based on L-PEI 25k afforded 65% cell viability at the 5:1 *w*/*w* mixing ratio, viability was only reduced to 58% with 10% TAC crosslinking. Overall, cell viability in the presence of PEI/DNA polyplexes was reduced by the incorporation of TAC, although in some cases not to a significant degree (Figure 3d).

Previous approaches to reduce toxicity have focused on modifications that have reduced both efficacy and toxicity [41,42]. We took the opposite approach, namely to enhance transfection efficiency in such a way that the amount of polymer introduced may be minimized, thereby reducing toxic effects. A 5:1 *w*/*w* mixing ratio of 10% crosslinked L-PEI 25k (II) afforded an increase in RLU detected by luciferase assay from 17 to 24 relative to unmodified PEI, while only modestly reducing cell viability (from 65% to 58%). The increase in efficacy without significant detrimental toxicity effects represents the advantage of PEI/TAC as a non-viral transfection agent. The toxicity recorded for 30% crosslinked L-PEI 25k (III) was prohibitive at the polymer/DNA mixing ratios required for efficacy, with only 41% cell viability. The 60% crosslinked L-PEI 25k (IV) was slightly less efficacious than II (23 RLU in luciferase assay compared to 24) and slightly more toxic (53% cell viability compared to 58%). Therefore, 10% crosslinked L-PEI 25k (II) was determined to be the best candidate for further evaluation, as it was the most efficacious PEI/TAC transfection agent without dramatically increasing toxicity.

### 3.5. Effects of PEI Molecular Architecture

The molecular architecture of PEI is known to have dramatic effects upon transfection efficiency [27,43]. We therefore sought to compare the effects of TAC crosslink with other PEI, either of lower molecular weight or with branching. We crosslinked both linear PEI of MW 5 kDa (L-PEI 5k, VI) and branched PEI of MW 1.3 kDa (B-PEI 1.3k, VIII) with 30% TAC. Crosslinking procedures were performed in the same manner as L-PEI 25k. These PEI/TACs formed nanoparticles upon dispersion in aqueous media (~100 nm) and positive zeta potentials, similar to crosslinked L-PEI 25k (Table 2).

**Figure 4:**
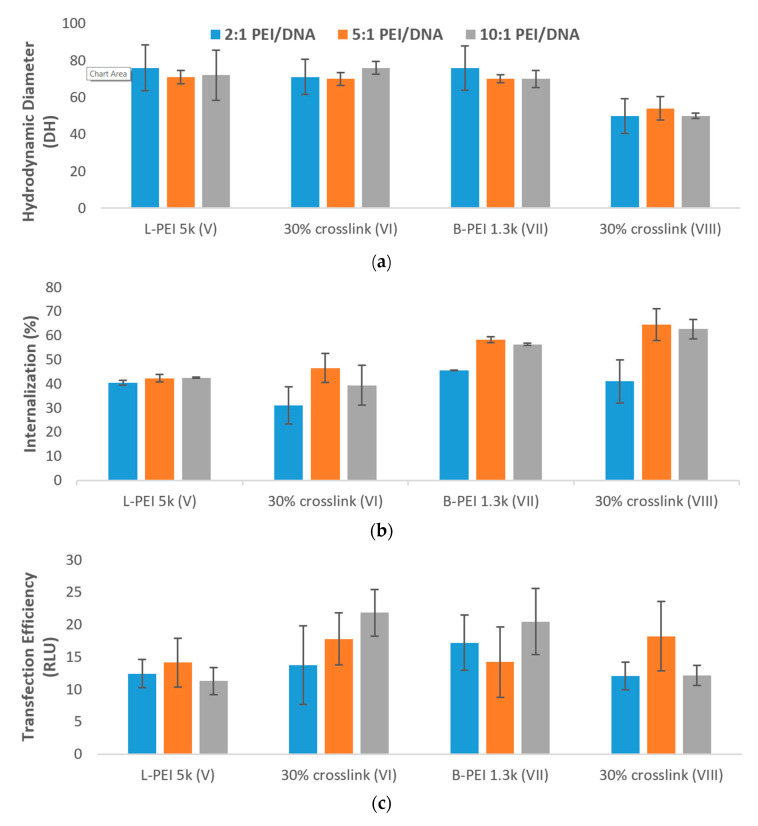

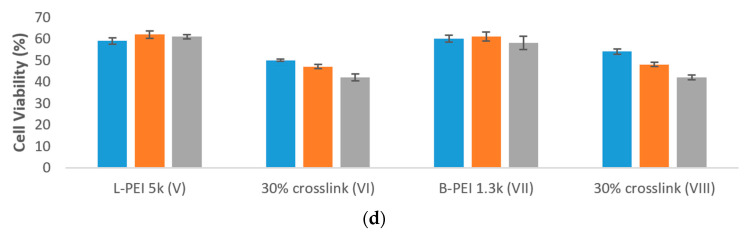
(**a**) Formation of PEI/TAC/DNA polyplexes based on L-PEI 5k and B-PEI 1.3k was confirmed by reduction in the hydrodynamic diameter of particles. Both unmodified PEI and PEI/TAC formed polyplexes with DNA, allowing for cellular uptake by endocytosis; (**b**) Internalization of DNA by PEI/TAC vectors based on L-PEI 5k and B-PEI 1.3k; (**c**) Transfection of DNA by PEI/TAC vectors based on L-PEI 5k and B-PEI 1.3k; (**d**) Cytotoxicity of PEI/TAC based on L-PEI 5k and B-PEI 1.3k.

The decreased particle size of 30% crosslinked B-PEI 1.3k (VIII, Figure 4a) afforded enhanced cellular uptake of PEI/TAC/DNA polyplexes (65% for 5:1 *w*/*w* mixing ratio) relative to unmodified PEI (58% uptake at the same ratio). The 30% crosslinked L-PEI 5k (VI), which did not display reduced particle size, displayed no advantage regarding cellular uptake relative to unmodified PEI, as neither afforded more than 47% uptake (Figure 4b).

Although PEI/TAC based on L-PEI 5k rendered no enhancement of cellular uptake, enhanced overall transfection was indeed observed. Unmodified L-PEI 5k afforded no more than 14 RLU in luciferase assay, whereas the 10% *w*/*w* mixing ratio of PEI/TAC:DNA afforded 22 RLU, greater than even that of the industry standard L-PEI 25k (17 RLU). As the polymer/DNA ratio was increased, overall transfection rose as well (14, 18, and 22 RLU for 2:1, 5:1 and 10:1 *w*/*w* mixing ratios, respectively, Figure 4c).

PEI/TAC based on B-PEI 1.3k, although displaying enhanced cellular uptake, did not improve overall transfection. This is likely due to the mechanism of endosomal escape, which requires ionizable secondary amines along the polymer chain. Some of the amine groups along the branched polymer chain were taken up for branching, and so the necessary proton sponge effect that destabilizes the endosome was hindered. Since the few remaining amine groups were used for crosslinking with TAC, few were left to enable endosomal escape, rendering overall transfection unenhanced relative to unmodified B-PEI 1.3k.

PEI/TAC based on L-PEI 5k and B-PEI 1.3k were also evaluated for their toxic effects (Figure 4d). As was observed for PEI/TAC based on L-PEI 25k, the incorporation of TAC into the polymer structure reduced cell viability in vitro. Cell viability of PEI/TAC of L-PEI 5k (VI) was reduced to 50% for the 2:1 polymer/DNA *w*/*w* mixing ratio, compared to 62% viability for unmodified L-PEI. Similarly, PEI/TAC of B-PEI 1.3k (VIII) resulted in 54% viability, lower than 61% for unmodified B-PEI. As the polymer/DNA mixing ratio was increased, viability tended to be reduced. Due to the limited enhancement of overall transfection as well as increasing toxicity, PEI/TAC polymers based upon L-PEI 25k may be considered the top candidates for further evaluation, and not those based on lower MW or branched PEI.

## 4. Conclusions

Cyclopropenium-containing nanoparticles displayed improved transfection efficiency over PEI, a common and efficient non-viral transfection vector. Formation of nanoparticles is a simple chemical transformation, where PEI is reacted at room temperature with tetrachlorocyclopropene. Complexation with plasmid DNA was spontaneous to form ~50 nm polyplexes that were found to be active in transfection of cells with low toxicity in vitro. In vivo studies are required for the confirmation of these agents as suitable transfecting agents.

## Figures and Tables

**Table 1:** Linear PEI of molecular weight (MW) 25 kDa (L-PEI 25k) was crosslinked with different amounts of TAC. Zeta potential and hydrodynamic diameter of polymers as 0.1% aqueous dispersions were evaluated.

Entry	PEI	Mol% Crosslinker	ζ Potential [mV]	D_H_ [nm]
I	25 kDa linear	0	n/a	n/a
II	25 kDa linear	10	16	98
III	25 kDa linear	30	7	93
IV	25 kDa linear	60	10	98

**Table 2:** L-PEI 5k and branched PEI of MW 1.3 kDa (B-PEI 1.3k) were crosslinked with 30% TAC. Zeta potential and hydrodynamic diameter of polymers as 0.1% aqueous dispersions were evaluated.

Entry	PEI	Mol% Crosslinker	ζ Potential [mV]	D_H_ [nm]
V	5 kDa linear	0	n/a	n/a
VI	5 kDa linear	30	11	109
VII	1.3 kDa branched	0	n/a	n/a
VIII	1.3 kDa branched	30	17	101

## Supplementary Materials

The following are available online at https://www.mdpi.com/1999-4923/12/8/768/s1, Figure S1: ^13^C NMR and IR spectra of crosslinked polymer, Figure S2: TGA of crosslinked polymer.
