# Supplementary material for: Cyclopropenium Nanoparticles and Gene Transfection in Cells
Source: Pharmaceutics. 2020 Aug 13;12(8):768. doi: 10.3390/pharmaceutics12080768 (PMC7465078; doi:10.3390/pharmaceutics12080768)
Supplement: Supplementary file 1 [file pharmaceutics-12-00768-s001.pdf]

pharmaceutics

MDPI

Supplementary Materials: Cyclopropenium Nanoparticles and Gene Transfection in Cells

Noam Y. Steinman, Luis M. Campos, Yakai Feng, Abraham J. Domb and Hossein Hosseinkhani

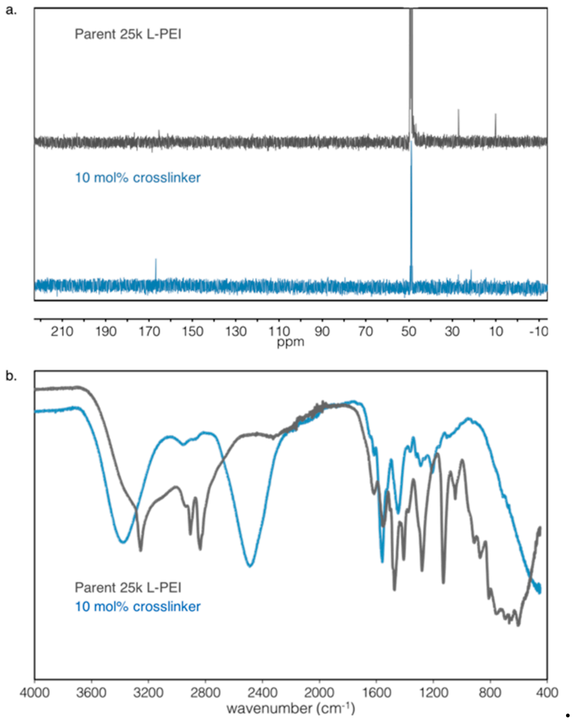

Figure S1. ^{13}C NMR (**a**) and IR (**b**) spectra of parent linear PEI compared to those of a crosslinked polymer. The appearance of aromatic carbon peaks (**a**) and disappearance of secondary amine absorbances (**b**) suggests in situ cyclopropenium ion formation and crosslinking with secondary amines.

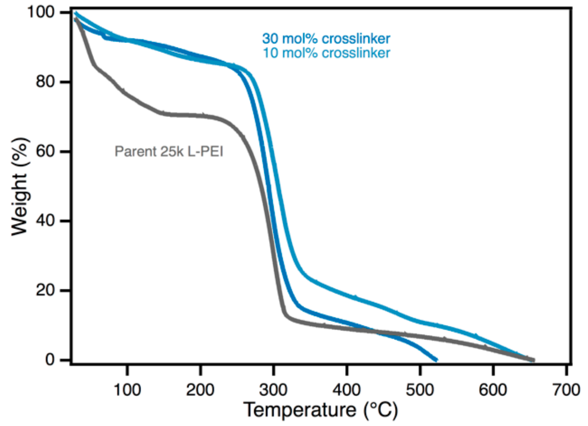

Figure S2. Thermal gravimetric analysis of crosslinked polymers. Cyclopropenium crosslinking of 25k L-PEI L-PEI provides enhanced stability below 300 °C. Crosslinking minimally changes thermal decomposition temperature. There is no change in thermal decomposition with increased crosslinking density.
